# Identification of the miRNA-mRNA regulatory network of small cell osteosarcoma based on RNA-seq

**DOI:** 10.18632/oncotarget.17208

**Published:** 2017-04-18

**Authors:** Lin Xie, Yedan Liao, Lida Shen, Fengdi Hu, Sunlin Yu, Yonghong Zhou, Ya Zhang, Yihao Yang, Dongqi Li, Minyan Ren, Zhongqin Yuan, Zuozhang Yang

**Affiliations:** ^1^ Bone and Soft Tissue Tumors Research Center of Yunnan Province, Department of Orthopedics, The Third Affiliated Hospital of Kunming Medical University, Tumor Hospital of Yunnan Province, Kunming, Yunnan 650118, China; ^2^ Department of Medical Oncology, The Third Affiliated Hospital of Kunming Medical University, Tumor Hospital of Yunnan Province, Kunming, Yunnan 650118, China

**Keywords:** small cell osteosarcoma, RNA-seq, microRNA, regulatory network

## Abstract

Small cell osteosarcoma (SCO) is a rare subtype of osteosarcoma characterized by highly aggressive progression and a poor prognosis. The miRNA and mRNA expression profiles of peripheral blood mononuclear cells (PBMCs) were obtained in 3 patients with SCO and 10 healthy individuals using high-throughput RNA-sequencing. We identified 37 dysregulated miRNAs and 1636 dysregulated mRNAs in patients with SCO compared to the healthy controls. Specifically, the 37 dysregulated miRNAs consisted of 27 up-regulated miRNAs and 10 down-regulated miRNAs; the 1636 dysregulated mRNAs consisted of 555 up-regulated mRNAs and 1081 down-regulated mRNAs. The target-genes of miRNAs were predicted, and 1334 negative correlations between miRNAs and mRNAs were used to construct an miRNA-mRNA regulatory network. Dysregulated genes were significantly enriched in pathways related to cancer, mTOR signaling and cell cycle signaling. Specifically, hsa-miR-26b-5p, hsa-miR-221-3p and hsa-miR-125b-2-3p were significantly dysregulated miRNAs and exhibited a high degree of connectivity with target genes. Overall, the expression of dysregulated genes in tumor tissues and peripheral blood samples of patients with SCO measured by quantitative real-time polymerase chain reaction corroborated with our bioinformatics analyses based on the expression profiles of PBMCs from patients with SCO. Thus, hsa-miR-26b-5p, hsa-miR-221-3p and hsa-miR-125b-2-3p may be involved in SCO tumorigenesis.

## INTRODUCTION

Osteosarcoma (OS) is a common primary bone tumor in young adults and adolescents [1], which is characterized by a high metastatic potential: approximately 20–25% of newly diagnosed patients harbor detectable lung-related metastases [2, 3]. Moreover, the distal femur and proximal tibia are frequent metastatic locations for OS. Despite recent improvements in therapeutic strategies, including surgical, radiotherapy and neoadjuvant chemotherapy, the 5-year survival of patients with OS remains below 70% [4].

Small-cell osteosarcoma (SCO) is a rare subtype of OS and accounts for less than 1% of OS cases [5]. SCO is highly aggressive and frequently found in the metaphyses of long bones, which results in a poor prognosis. Histologically, SCO presents as small, round, primitive, and undifferentiated cells with osteoid production [6]. Moreover, SCO does not respond to radiotherapy and is more aggressive than other subtypes of OS [7]. Thus, novel therapeutic strategies for patients with SCO are urgently needed.

The etiologic factors and mechanism underlying the pathogenesis of OS are currently unclear. An increasing number of studies show that microRNAs (miRNAs) play important roles in the tumorigenesis and development of OS. miRNAs are small non-coding RNA molecules (18–25 nt) that increase or repress the expression of target genes [8]. Specifically, the down-regulation of miR-133a and miR-539 are associated with an unfavorable prognosis for patients suffering from OS [9]. Moreover, miR-664 acts as an oncogene and promotes the proliferation of OS cells by down-regulating the expression of FOXO4. In addition, miR-664 promotes OS cell invasion and migration by suppressing the expression of SOX7 [10, 11]. Osteopontin is a phosphorylated glycoprotein involved in the invasion of OS cells, and the suppression of miR-4262 in OS cells promotes osteopontin-mediated cancer invasion [12]. Furthermore, the down-regulation of miR-26a and up-regulation of miR-27a reportedly contribute to the aggressiveness of OS, and low miR-26a expression and high miR-27a expression are associated with advanced TNM stage, tumor grade, and distant metastasis in patients with OS [13].

SCO is an exceedingly rare subtype of OS associated with a poor prognosis. However, the mechanism underlying the pathogenesis of SCO is unclear, and previous studies of SCO are case reports due to the rarity of this disease. In this study, high-throughput RNA sequencing was performed to obtain the miRNA and mRNA expression profiles of peripheral blood mononuclear cells (PBMCs) from patients with SCO to identify genes that are differentially expressed in these patients compared with healthy individuals and explore potential diagnostic biomarkers for SCO.

## RESULTS

### Genes dysregulated in patients with SCO compared with healthy individuals

Blood samples from 3 patients with SCO and 10 healthy control individuals were subjected to high-throughput RNA sequencing; mRNA reads were used to align to the UCSC human reference genome (hg.19), and miRNA reads were matched to miRBase database. Differences in the expression levels of miRNA and mRNA between samples from patients with SCO and healthy individuals were then analyzed.

Compared with healthy individuals, 37 dysregulated miRNAs, including 27 up-regulated and 10 down-regulated miRNAs, were identified in the PBMCs of patients with SCO patients, as shown in Table 1. Moreover, 1636 dysregulated mRNAs, including 555 up-regulated mRNAs and 1081 down-regulated mRNAs, were also identified in SCO. The top 20 significantly up-regulated and top 20 down-regulated mRNAs are listed in Table 2.

**Table 1:** Differentially expressed miRNAs in SCO compared with normal controls

miRNA	log_2_FC	*P*-value
**Up-regulated miRNAs**		
hsa-miR-4676-5p	4.103148	0.008024354
hsa-miR-6842-5p	3.101667	0.008024354
hsa-miR-580-5p	2.882593	0.004165488
hsa-miR-7850-5p	1.607435	0.005552087
hsa-miR-5706	1.400998	0.002164524
hsa-miR-627-5p	1.384528	0.003693182
hsa-miR-6868-3p	1.296777	0.004165102
hsa-miR-19a-5p	1.268304	0.007646879
hsa-miR-548e-3p	1.028317	0.001442817
hsa-miR-21-5p	0.933647	0.007984057
hsa-miR-191-5p	0.885862	0.000803189
hsa-miR-3605-5p	0.74933	0.009822637
hsa-miR-26b-5p	0.741	0.000629361
hsa-let-7a-3p	0.739606	0.002230048
hsa-miR-556-3p	0.712671	0.008436748
hsa-miR-548j-3p	0.690548	0.000462576
hsa-miR-500a-3p	0.637856	0.004488069
hsa-miR-5690	0.63544	0.006641347
hsa-miR-221-5p	0.600366	0.004591464
hsa-miR-6802-3p	0.559877	0.004209508
hsa-miR-3174	0.339832	0.0009132
hsa-miR-7-1-3p	0.272796	0.001506044
hsa-miR-4781-3p	0.265409	0.00649929
hsa-miR-4511	0.171781	0.002387782
hsa-miR-6859-5p	0.137966	0.001116084
hsa-miR-99b-3p	0.077028	0.006497498
hsa-miR-1307-3p	0.058939	0.001262399
**Down-regulated miRNAs**		
hsa-miR-4685-3p	−2.02683	0.006488539
hsa-miR-125b-2-3p	−1.84345	0.008826503
hsa-miR-379-3p	−1.16001	6.49E−07
hsa-miR-656-3p	−0.54022	0.001139873
hsa-miR-222-3p	−0.49502	0.003284816
hsa-miR-570-3p	−0.25872	0.009221198
hsa-miR-576-5p	−0.15013	0.006721753
hsa-miR-221-3p	−0.12721	0.006039399
hsa-miR-1260b	−0.04075	0.000537561
hsa-miR-1284	−0.00496	0.001779152

SCO: small-cell osteosarcoma; FC: fold change.

**Table 2:** Differentially expressed mRNAs in SCO compared with normal controls

Gene symbol	Log_2_FC	*P*-value
up-regulated mRNAs		
TINCR	3.028985	0.039245
SMIM1	2.86932	0.027698
RBBP8	2.690018	0.015199
SORCS2	2.653466	0.000205
FAM89A	2.633881	0.021369
CABP7	2.53465	0.011636
SPATA24	2.520196	0.043857
PGLYRP2	2.334439	0.046067
DNASE1L2	2.309282	0.011064
LOC101928111	2.292573	0.012952
ERGIC2	2.258767	0.022173
PTGFRN	2.247947	0.02845
PROS1	2.221589	0.017288
TIMP3	2.221582	0.017287
RAB3A	2.207956	0.046707
C16orf91	2.188473	0.045652
FAM83D	2.046992	0.023751
GPRC5C	2.044382	0.033657
LAMC3	2.035623	0.042842
PLOD2	1.995595	0.034861
Down-regulated mRNAs		
TICAM2	−3.38105	0.018968
MUC20	−2.46798	0.008805
MS4A1	−2.30667	0.006431
LOC100505716	−2.26707	0.044691
SLFN12L	−2.24573	0.033332
TM4SF19	−2.22329	0.03167
HEMGN	−2.22149	0.035928
ARHGEF10	−2.21973	0.026584
LOC100129215	−2.20143	0.029742
POLR2K	−2.17318	0.02433
FASLG	−2.17244	0.028758
ZNF41	−2.16603	0.024358
PLEKHA8	−2.14986	0.034366
RSAD2	−2.11035	0.043712
BMS1P5	−2.07825	0.01964
TTC21B	−2.06803	0.039198
NCR3LG1	−2.06803	0.049214
ATP6V1G2-DDX39B	−2.04579	0.044986
NUP107	−2.03082	0.042533
RIF1	−1.997	0.008844

SCO: small-cell osteosarcoma.

### GO annotation of dysregulated genes in SCO

**Figure 1:**
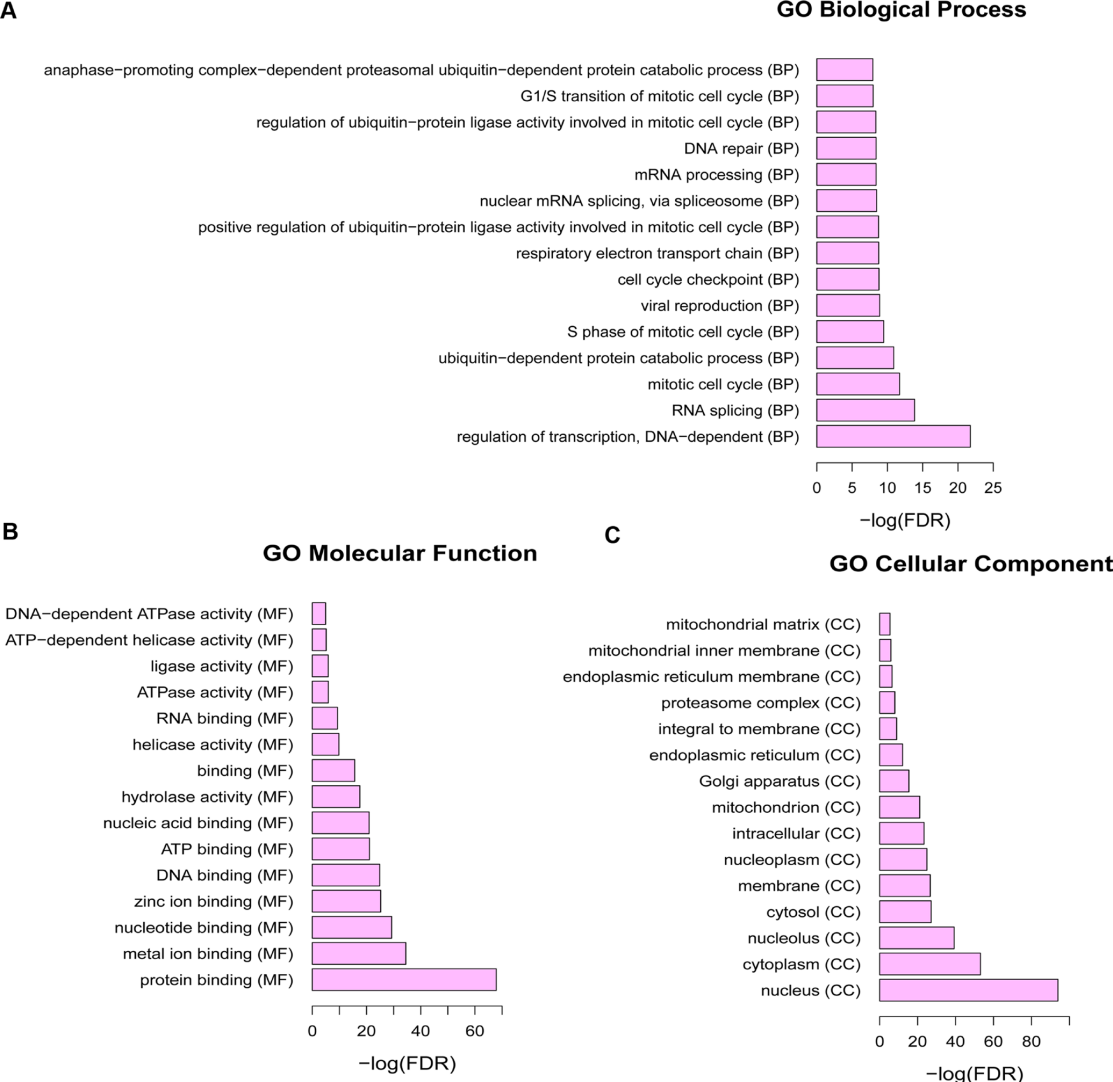
The Gene Ontology term enrichment of dysregulated mRNAs in SCO FDR: false discovery rate. (**A**): Biological processes of Gene Ontology terms; (**B**): molecular functions of Gene Ontology terms; (**C**): cellular components of Gene Ontology terms.

1636 dysregulated genes in SCO were performed to GO annotation to obtain the biological roles. GO terms with FDR < 0.05 were considered as significant enrichment. Regulation of transcription, DNA-dependent (GO: 0006355, FDR = 1.77E-22), RNA splicing (GO: 0008380, FDR = 1.41E-14) and mitotic cell cycle (GO: 0000278, FDR = 1.89E-12) were the most significant enrichments of biological process (Figure 1A); protein binding (GO: 0005515, FDR = 1.32E-68), metal ion binding (GO: 0046872, FDR = 3.16E-35) and nucleotide binding (GO: 0000166, FDR = 5.40E-30) were the highest enrichment of molecular function (Figure 1B); nucleus (GO: 0005634, FDR = 1.15E-94), cytoplasm (GO: 0005737, FDR = 7.67E-54) and nucleolus (GO: 0005730, FDR = 5.86E-40) was the highest enrichment of cellular component, as Figure 1C shown.

### KEGG pathway annotation of dysregulated genes in SCO

KEGG is a knowledge database for the systematic functional analysis of genes to connect genomic information with higher functional information by computerizing current knowledge on cellular processes and standardizing gene annotations. Thus, KEGG analyses were performed to assign pathways and functionally classify genes that are dysregulated in SCO. An FDR < 0.05 was defined as significant KEGG pathway enrichment. As shown in Table 3, pathways in cancer, mTOR signaling, oxidative phosphorylation, cell cycle, apoptosis, and DNA replication were significantly enriched KEGG pathways.

**Table 3:** KEGG analysis of dysregulated mRNAs in SCO

KEGG ID	Items	No.	FDR	Genes
Kegg: 00190	Oxidative phosphorylation	22	1.23E-06	*NDUFB4, ATP5J, ATP6V1F, ATP6V1G1, NDUFA2, UQCRC1, COX6A1, CYC1, COX6B1,NDUFB10, COX5B, ATP5J2, ATP5H, ATP5E, COX8A, NDUFA3, COX7A2, SDHD, UQCR11,NDUFA11, UQCRQ, ATP5B*
Kegg: 05221	Acute myeloid leukemia	11	0.000311	*MTOR, BAD, PIK3CB, RPS6KB1, RUNX1, PIM2, PIK3R1, MAP2K2, RPS6KB2, AKT2, PIM1*
Kegg: 04210	Apoptosis	13	0.000714	*BAD, CASP7, EXOG, PIK3CB, CASP10, BIRC3, FASLG, IRAK1, PIK3R1, PRKX, BAX, AKT2, ATM*
Kegg: 03030	DNA replication	8	0.000915	*POLA1, RFC2, RFC1, MCM6, POLD4, PRIM1, PCNA, RFC4*
Kegg: 05200	Pathways in cancer	29	0.001249	*WNT10A, MTOR, MSH2, PTCH1, TRAF5, BAD, STAT1, DAPK3, BIRC5, PIK3CB, LAMA2, PRKCG,BIRC3, RUNX1, FASLG, MSH6, PIK3R1, FZD4, VHL, LAMC3, MAP2K2, FZD3, BAX, FGF11, DAPK1, PDGFB, AKT2, TCEB2, CBLB*
Kegg: 04012	ErbB signaling pathway	12	0.002552	*MTOR, BAD, PIK3CB, PRKCG, RPS6KB1, PAK4, PIK3R1, SHC1, MAP2K2, RPS6KB2, AKT2, CBLB*
Kegg: 04150	mTOR signaling pathway	8	0.009348	*MTOR, PIK3CB, RPS6KB1, TSC1, PIK3R1, TSC2, RPS6KB2, AKT2*
Kegg: 03018	RNA degradation	9	0.010472	*SKIV2L2, CNOT7, PAN2, TTC37, DHX36, EXOSC9, HSPD1, EXOSC8, LSM3*
Kegg: 04110	Cell cycle	13	0.01212	*CDC14A, FZR1, ANAPC1, CDC23, ATR, ANAPC4, CCNB1, MCM6, CUL1, CDC14B, PCNA, E2F4, ATM*
Kegg: 04622	RIG-I-like receptor signaling pathway	9	0.014533	*DHX58, SIKE1, OTUD5, CASP10, DDX3Y, RNF125, ATG12, CYLD, IFIH1*
Kegg: 05211	Renal cell carcinoma	9	0.014533	*PTPN11, PIK3CB, PAK4, PIK3R1, VHL, MAP2K2, PDGFB, AKT2, TCEB2*
Kegg: 05220	Chronic myeloid leukemia	9	0.017915	*BAD, PTPN11, PIK3CB, RUNX1, PIK3R1, SHC1, MAP2K2, AKT2, CBLB*
Kegg: 05215	Prostate cancer	10	0.018952	*MTOR, BAD, CREB3L2, PIK3CB, PDGFD, CREB3, PIK3R1, MAP2K2, PDGFB, AKT2*
Kegg: 05210	Colorectal cancer	8	0.019385	*MSH2, BAD, BIRC5, PIK3CB, MSH6, PIK3R1, BAX, AKT2*
Kegg: 05214	Glioma	8	0.020495	*MTOR, PIK3CB, PRKCG, PIK3R1, SHC1, MAP2K2, PDGFB, AKT2*
Kegg: 00030	Pentose phosphate pathway	5	0.020611	*PGLS, G6PD, TKTL1, RPE, PGD*
Kegg: 03020	RNA polymerase	5	0.022375	*POLR1A, POLR3B, POLR2E, POLR2K, POLR3F*
Kegg: 04062	Chemokine signaling pathway	16	0.022417	*CXCL6, STAT1, XCL2, XCL1, TIAM1, PIK3CB, CXCR5, GNG3, PIK3R1, SHC1, PRKX, CCR5,RASGRP2, AKT2, CCR6, CXCR6*
Kegg: 05145	Toxoplasmosis	12	0.02264	*BAD, STAT1, PIK3CB, LAMA2, CD40, BIRC3, HLA-DOB, IRAK1, PIK3R1, LAMC3, CCR5, AKT2*
Kegg: 04650	Natural killer cell mediated cytotoxicity	12	0.024279	*PTPN11, KLRK1, HLA-A, PIK3CB, NFATC3, PRKCG, SH2D1A, FASLG, KLRD1, PIK3R1,SHC1, MAP2K2*
Kegg: 04350	TGF-beta signaling pathway	9	0.027483	*RPS6KB1, PPP2R1B, PPP2CB, PPP2R1A, BMP8B, RPS6KB2, CUL1, ACVR2A, E2F4*
Kegg: 05218	Melanoma	8	0.030816	*BAD, PIK3CB, PDGFD, PIK3R1, MAP2K2, FGF11, PDGFB, AKT2*
Kegg: 04510	Focal adhesion	16	0.031225	*BAD, ITGA4, PIK3CB, ACTG1, LAMA2, PDGFD, PRKCG, BIRC3, PAK4, ITGA10, PIK3R1, SHC1,LAMC3, PPP1CA, PDGFB, AKT2*
Kegg: 04310	Wnt signaling pathway	13	0.033068	*WNT10A, NFATC3, PRKCG, PRICKLE1, CACYBP, PPP2R1B, CSNK2A1, PPP2CB, PPP2R1A,FZD4, PRKX, FZD3, CUL1*

SCO: small-cell osteosarcoma; FDR: false discovery rate; No.: number of genes in pathway.

### The regulatory network of miRNA target genes in SCO

**Figure 2:**
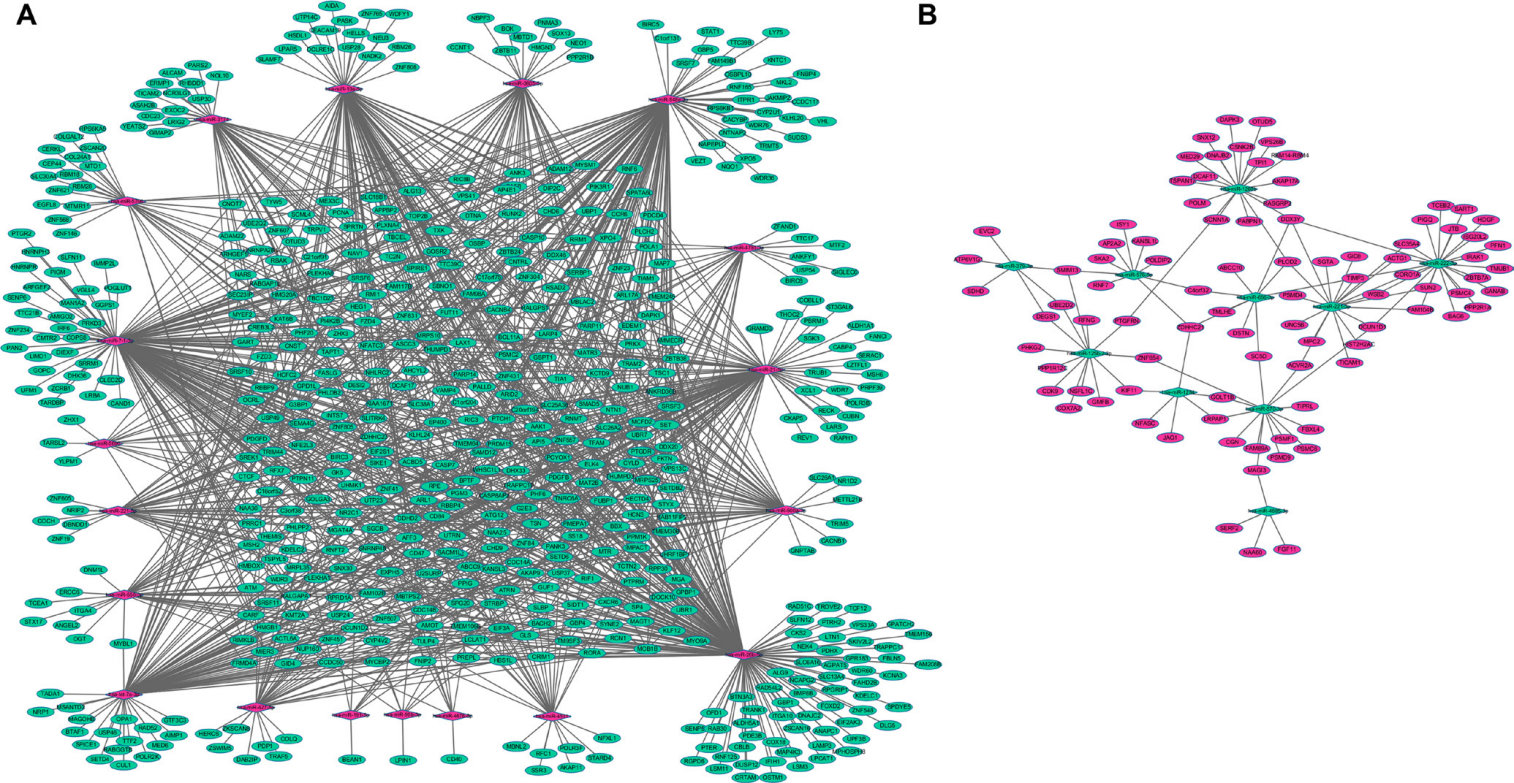
miRNA-mRNA interaction network in SCO (**A**): Interaction network between up-regulated miRNAs and down-regulated mRNAs; (**B**): interaction network between down-regulated miRNAs and up-regulated mRNAs. Circular nodes represented mRNAs, and diamond nodes represented miRNAs. Green and red colors represented down-regulation and up-regulation, respectively. Solid lines indicated interaction associations between miRNAs and mRNAs.

The target-genes of dysregulated miRNAs in SCO were predicted based on the miRWalk database. Genes that were both differentially expressed and targets of dysregulated miRNAs were candidates to construct the miRNA-mRNA network. Specifically, 1334 miRNA-target gene pairs with reverse correlation retrieved by the miRWalk database, including 1076 pairs of up-regulated miRNAs and 93 pairs of down-regulated miRNAs, were used to construct the miRNA-mRNA regulatory network and visualized using the Cytoscape software (Figure 2). The target-genes of 8 miRNAs, including hsa-miR-548j-3p, hsa-miR-6859-5p, hsa-miR-1307-3p, hsa-miR-6868-3p, hsa-miR-580-5p, hsa-miR-6802-3p, hsa-miR-7850-5p and hsa-miR-6842-5p, were not available in the miRWalk database. In the network, the up-regulated miRNAs hsa-miR-26b-5p, hsa-miR-1-1-3p and hsa-miR-548e-3p, exhibited the highest connectivity with target-genes and regulated 197, 162 and 141 target-genes, respectively. Moreover, the down-regulated miRNAs hsa-miR-222-3p, hsa-miR-1260b and hsa-miR-221-3p exhibited the highest connectivity to target genes and regulated 23, 19 and 18 target-genes, respectively (Figure 2).

### Verification of the expression level of dysregulated genes in tumor tissues and peripheral blood samples from patients with SCO

**Figure 3:**
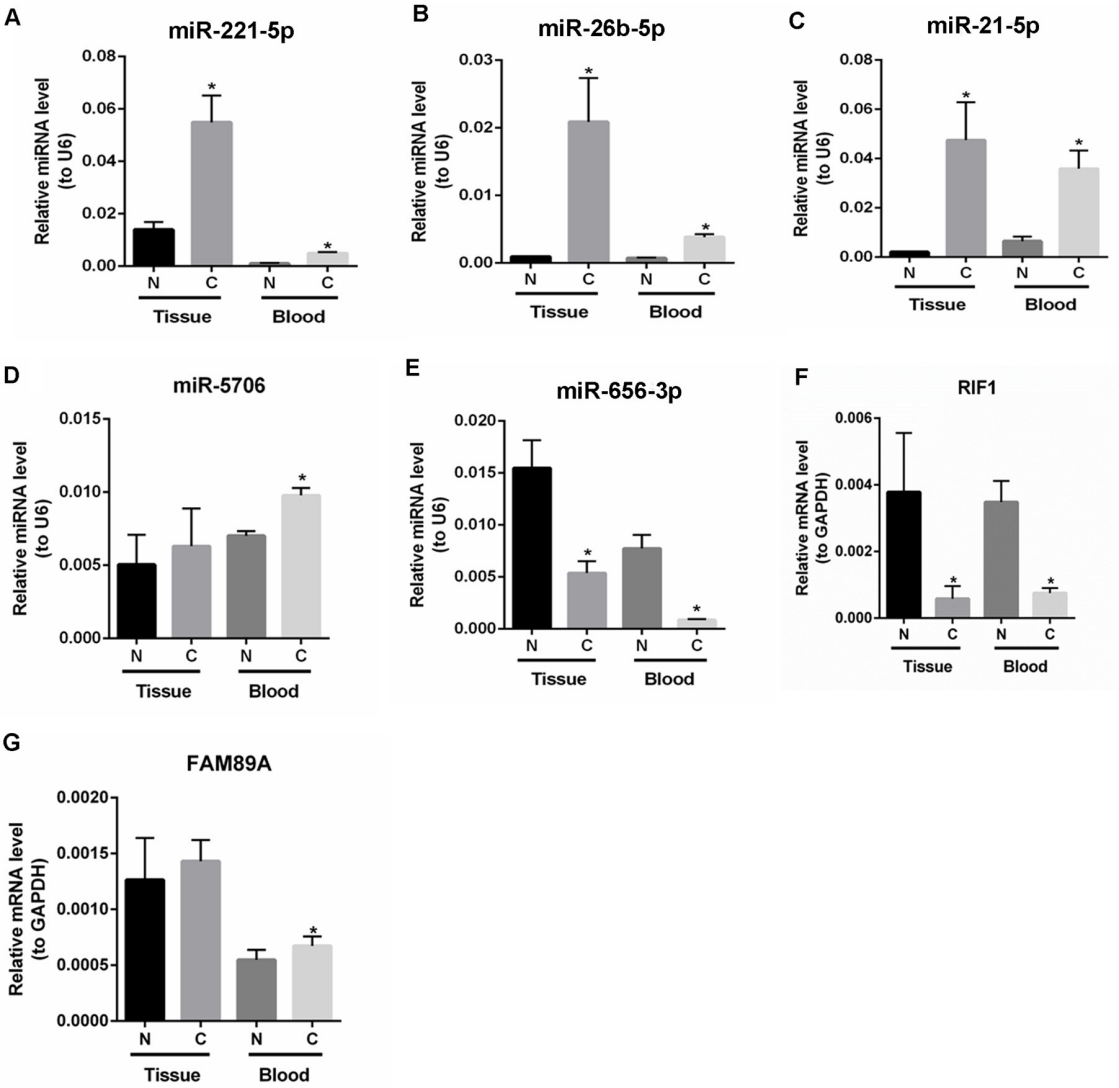
The expression levels of candidate mRNAs and miRNAs in peripheral blood samples and tumor tissues from patients with SCO were verified by qRT-PCR (**A**): hsa-miR-221-5p; (**B**): hsa-miR-26b-5p; (**C**): hsa-miR-21-5p; (**D**): hsa-miR-5706; (**E**): hsa-miR-656-3p; (**F**): RIF1; (**G**): FAM89A. Blood N: peripheral blood samples of healthy individuals; Blood C: peripheral blood samples of patients with SCO; Tissue N: adjacent non-tumor tissues of SCO patients; Tissue C: SCO tumor tissues. At least three independent experiments were performed for the statistical evaluation.

To verify the RNA-seq data, the expression levels of differentially expressed miRNAs and mRNAs in tumor tissues and peripheral blood samples from patients with SCO were quantified by qRT-PCR. As shown in Figure 3A–3C, hsa-miR-221-5p, hsa-miR-26b-5p and hsa-miR-21-5p were significantly up-regulated in both tumor tissues and peripheral blood samples from patients with SCO. The expression of miR-5706 was significantly up-regulated in peripheral blood samples from patients with SCO patients with healthy individuals, but its expression did not significantly differ between SCO tumors and adjacent non-tumor tissues (Figure 3D). As shown in Figure 3E and 3F, miR-656-3p and RIF1 were significantly down-regulated in both tumor tissues and peripheral blood samples from patients with SCO, whereas FAM89A was significantly up-regulated in peripheral blood samples from patients with SCO compared with healthy individuals, but its expression did not significantly differ between SCO tumors and adjacent non-tumor tissues (Figure 3G). In general, qRT-PCR data were consistent with our bioinformatics analyses.

## DISCUSSION

miRNAs are endogenous, small non-coding RNAs that participate in the regulation of diverse cellular processes, including cell growth, cell differentiation, apoptosis and the immune response, by binding to the 3′ untranslated region of certain subsets of messenger RNAs. A growing body of evidence demonstrates that the abnormal expression of miRNAs might serve as a potential diagnostic and prognostic biomarker for cancers [14].

In our study, the mRNA and miRNA expression profiles of peripheral blood samples from patients with SCO were obtained via RNA sequencing. Dysregulated mRNAs and miRNAs were identified *in* silico, and the expression levels of candidate mRNAs and miRNAs in both tumor tissues and peripheral blood samples from patients with SCO were verified by qRT-PCR. In general, the expression status of candidate mRNAs and miRNAs in peripheral blood samples of SCO patients were consistent with our bioinformatics analysis based on the expression profiling of PBMCs of SCO patients. Moreover, the expression levels of candidate mRNAs and miRNAs were also detected in SCO tumor tissues and adjacent non-tumor tissues. qRT-PCR results of candidate mRNAs and miRNAs had the similarity expression status in tumor tissues of OS patients based on bioinformatics analyses.

hsa-miR-26b-5p was significantly up-regulated in the PBMCs from patients with SCO compared to healthy control patients. In the miRNA-target gene interaction network, miR-26b-5p exhibited the highest connectivity with miRNAs and regulated the expression of 197 target-genes. Specifically, miR-26b-5p is reportedly down-regulated in OS tissue and cells [15, 16] and inhibits OS cell proliferation, migration, invasion and apoptosis by down-regulating the expression of PFKFB3 [15]. Moreover, miR-26b inhibits OS cell metastasis by suppressing the expression of CTGF and Smad1 [17]. Huaier (*Trametes robiniophila murris*) is a fungus used in traditional Chinese medicine and suppresses cell proliferation and induces apoptosis in human lung cancer cells by up-regulating miR-26b-5p [18]. miR-26b plays important roles in the proliferation and metastasis of various cancer types, such as epithelial ovarian carcinoma, hepatocellular carcinoma and prostate cancer [19–21]. FASLG is one of top 20 down-regulated genes in SCO and targeted by miR-26b. Moreover, FASLG encodes Fas ligand, which is a member of tumor necrosis factor superfamily. FAS then triggers cell apoptosis by binding to FASLG. The FAS/FASLG signaling pathway is essential for immune system regulation, including activation-induced T cell death and cytotoxic T lymphocyte-induced cell death. In our study, miR-26b-5p was up-regulated in the PBMCs of patients with SCO and targeted FASLG, which may play a key role in the immune response to SCO.

Furthermore, hsa-miR-221-3p was significantly down-regulated in the PBMCs from patients with SCO. In the miRNA-mRNA regulatory network, miRNA-221 exhibited high connectivity with several mRNAs and targeted 18 mRNAs, and the expression levels of miR-221 in tumor tissue, cell lines and the sera from patients with OS are reportedly dramatically up-regulated. Thus, serum miR-221 may serve as a diagnostic and prognostic marker for OS [22, 23]. Moreover, miR-221 enhances the proliferation, invasion and migration of OS cells by suppressing PTEN expression [22]. TIMP3 is one of top 20 up-regulated genes in SCO and targeted by miR-221. TIMP3 encodes TIMP metallopeptidase inhibitor 3, an inhibitor of matrix metalloproteinases, which are involved in the degradation of the extracellular matrix. TIMP3 is hypermethylated in OS tumor tissues compared with normal tissues [24] and has been identified as a tumor suppressor that plays essential roles in the inhibition of tumor angiogenesis. The down-regulation of miR-221/222 significantly increases the expression of TIMP3 and enhances the sensitivity of breast cancer cells to tamoxifen [25]. TIMP3 expression also predicts favorable survival in HCC [26]. In our study, miR-221-3p was down-regulated and targeted TIMP3 in the PBMCs of patients with SCO, which suggested that miR-221-3p and TIMP3 play essential roles in the immune response to SCO.

hsa-miR-125b-2-3p is one of the top 2 significantly down-regulated miRNAs and targeted 13 mRNAs, including PTGFRN (one of the top 20 up-regulated mRNAs) in the miRNA-mRNA regulatory network of SCO. PTGFRN encodes prostaglandin F2 receptor inhibitor, and the expression of PTGFRN correlates with the metastatic status of human OS [27]. However, the biological functions of miR-125b-2-3p and PTGFRN are unclear and need to be elucidated.

Pathways in cancer, mTOR signaling and oxidative phosphorylation were significantly enriched in the KEGG pathway analysis of dysregulated mRNAs identified in the PBMCs of patients with SCO compared to healthy individuals. Dysregulated pathways in cancer contribute to aberrant cell growth, cell death and cell motility in various cancer types, including colorectal cancer, pancreatic cancer, glioma, basal cell carcinoma, renal cell carcinoma, prostate cancer and melanoma [28, 29]. Moreover, a series of articles report that abnormal mTOR signaling plays key roles in OS tumorigenesis and therapy [30], and arsenic sulfide promotes cell apoptosis and autophagy by suppressing Akt/mTOR signaling in OS [31]. In addition, cucurbitacin E also inhibits OS cell growth and invasion by suppressing PI3K/AKT/mTOR signaling [32], whereas a lack of oxidative phosphorylation prevents cell apoptosis in colorectal cancer and OS. Accordingly, PSB-603 (A2b adenosine receptor antagonist) increases colorectal cancer cell death by promoting oxidative phosphorylation and ROS production [33]. A lack of oxidative phosphorylation also decreases susceptibility to apoptosis in OS [34]. In addition, cell cycle, apoptosis, and DNA replication pathways were significantly enriched in our study. Based on the aforementioned studies, the identified abnormally expressed mRNAs might play vital roles in OS tumorigenesis by regulating these enriched KEGG pathways.

The expression levels of candidate miRNAs and mRNAs in peripheral blood samples from patients with SCO were verified by qRT-PCR. In general, the qRT-PCR results were consistent with the RNA sequencing and bioinformatics analyses. However, our study was also subject to limitations. First, the SCO tumor sample size for the RNA sequencing and qRT-PCR validation analyses were small due to the rarity of SCO. Second, the expression levels of candidate miRNAs contradicted previously published results. Thus, the biological roles of these miRNAs need to be explored *in vivo* and *in vitro* to elucidate their roles in the tumorigenesis of SCO.

In conclusion, we constructed an miRNA-mRNA regulatory network by identifying miRNAs and target genes that are differentially expressed in the PBMCs of patients with SCO compared to healthy individuals. In this regulatory network, hsa-miR-26b-5p, hsa-miR-221-3p and hsa-miR-125b-2-3p were significantly dysregulated and exhibited high connectivity with target genes. Thus, these miRNAs may play essential roles in the initiation and development of SCO, but their biological roles in SCO tumorigenesis require further exploration. Moreover, a clinical study based on a large cohort of patients with SCO is essential to validate the diagnostic value of these miRNAs.

## MATERIALS AND METHODS

### Sample isolation and characterization

Thirteen patients treated at the Third Affiliated Hospital of Kunming Medical University hospital, including 3 patients with SCO and 10 healthy control individuals, were enrolled in our study. Ten milliliters of peripheral blood was obtained from each of patients, and PMBCs were isolated. The total RNA was then extracted from the PMBCs using TRIzol reagent (Invitrogen, Carlsbad, CA, USA). Our study was approved by the Third Affiliated Hospital of Kunming Medical University hospital, and informed written consent was obtained from all patients. Our study was conducted in accordance with the Declaration of Helsinki.

### Transcriptome library preparation and sequencing

The Illumina TruSeq RNA sample Prep Kit (Illumina, Inc., San Diego, CA, USA) was used to prepare the mRNA library according to the manufacturer's protocol. Oligo(dT) beads were used to isolate polyA mRNA from total RNA, and the mRNA was cleaved into short fragments for random hexamer priming to synthesize first-strand cDNA, followed by the second-strand cDNA synthesis and end repair. Next, the short fragments were connected with sequencing adapters. After PCR amplification, the enriched cDNA libraries were sequenced using an Illumina HiSeq 2500 (Illumina, Inc., San Diego, CA, USA). For miRNA sequencing, the miRNAs were isolated, and adapters were added. The library was constructed by using TruSeq Small RNA Library Preparation Kits (Illumina, Inc., San Diego, CA, USA).

### Data analyses

The raw RNA-sequencing reads were stored in FASTQ format. The raw reads containing adapter sequences and low-quality sequences (reads with ambiguous bases ‘N’) were removed using FASTx-tool SeqPrep (https://github.com/jstjohn/SeqPrep) and Sickle (https://github.com/najoshi/sickle). Clean reads were aligned with the UCSC human reference genome (build hg19) using TopHat v1.3.1 [35]. Sequences were matched to miRNAs using the miRDeep2 tool and miRBase (release 21) (http://www.mirbase.org/). Aligned read files were then processed by Cufflinks v1.2.1 [36], which measured the relative expression of the genes with the normalized RNA-Seq fragment counts. Fragments per kilobase of exon model per million mapped reads (FPKM) were used to present the expression level of gene. The transcripts differentially expressed in SCO were identified, and *P*-values < 0.05 and < 0.01 indicated differentially expressed mRNA and miRNA, respectively.

### Functional annotation of differentially expressed genes

The biological roles of differentially expressed genes were predicted using the GeneCoDis3 (http://genecodis.cnb.csic.es/analysis) database to describe the gene ontology (GO) and Kyoto encyclopedia of genes and genomes (KEGG) pathways [37, 38]. An FDR < 0.05 was set as the cut-off for selecting significantly enriched GO terms and KEGG pathway.

### Construction of miRNA-target gene network

The target-genes of differentially expressed miRNAs were predicted using the miRWalk database (http://www.umm.uni-heidelberg.de/apps/zmf/mirwalk/), in which the correlations between target-genes and miRNAs have been experimentally confirmed *in vivo* and *in vitro* [39]. We used 6 algorithms, RNA22, miRanda, miRDB, miRWalk, PICTAR2 and TargetScan, to predict the target-genes of miRNAs; if more than 4 of 6 algorithms predicted the same gene for an miRNA, the gene was identified as a target-gene of that miRNA [39]. The interaction network of differentially expressed miRNAs and target-genes were constructed using the Cytoscape software (http://cytoscape.org) [40].

### Quantitative real-time polymerase chain reaction (qRT-PCR)

To validate the expression levels of dysregulated genes in SCO, tumor tissues and peripheral blood samples were obtained from other 3 patients with SCO and 3 healthy individuals. Total RNA was extracted from tissues samples and peripheral blood samples using TRIzol reagent (Invitrogen, CA, USA) according to the manual instructions. The ReverTra Ace qPCR RT Master Mix Kit (TOYOBO, Shanghai, China) was used to synthesize cDNA, and qRT-PCR reactions were performed using SYBR^®^ FAST qPCR Kits (KAPA bio, Boston, USA) on a LightCycler 480 (Roche Indianapolis, IN, USA). U6 and GAPDH were used as internal controls for miRNA and mRNA detection, respectively. The relative expression levels of target genes were calculated using ΔCT method, and the mean ± standard deviation and independent samples *t*-test were used in the statistical analysis. *P* < 0.05 was considered significant. The PCR primers used are shown in Supplementary Table 1.

## SUPPLEMENTARY MATERIALS TABLE


