# Supplementary material for: Identification of the miRNA-mRNA regulatory network of small cell osteosarcoma based on RNA-seq
Source: Oncotarget. 2017 Apr 18;8(26):42525–36. doi: 10.18632/oncotarget.17208 (PMC5522085; doi:10.18632/oncotarget.17208)
Supplement: Supplementary file 1 [file oncotarget-08-42525-s001.pdf]

Identification of the miRNA-mRNA regulatory network of small cell osteosarcoma based on RNA-seq

Supplementary Materials

Supplementary Table 1: The primer list of genes for qRT-PCR detection

Genes	Primer sequence	
RIF1	Forward sequence:	CTCAGTATAGTCAGGAAGAGCCT
	Reverse sequence:	TCAGCCATACCACAGTCTTCCG
FAM89A	Forward sequence:	CTGCACTTACGCTCTGGAGAAC
	Reverse sequence:	GAGGAGACAGGCAGTGACAAGT
GAPDH	Forward sequence:	CTTAGCACCCCTGGCCAAG
	Reverse sequence:	GATGTTCTGGAGAGCCCCG
miR-656-3p	Forward sequence:	AATATTATACAGTCAACCTCT
miR-221-5p	Forward sequence:	ACCTGGCATACAATGTAGATTT
miR-21-5p	Forward sequence:	TAGCTTATCAGACTGATGTTGA
miR-26b-5p	Forward sequence:	TTCAAGTAATTCAGGATAGGT
miR-5706	Forward sequence:	TTCTGGATAACATGCTGAAGCT
U6	Forward sequence:	CTCGCTTCGGCAGCACA
	Reverse sequence:	AACGCTTCACGAATTTGCGT
